# Demonstration of Pattern Size Effects on Hydrophobic Nanocellulose Coatings with Regular Micron-Sized Island-like Geometrical Domains Created by Femtosecond Laser Micromachining

**DOI:** 10.3390/mi16030289

**Published:** 2025-02-28

**Authors:** Pieter Samyn, Patrick Cosemans, Olivier Malek

**Affiliations:** 1 Department of Innovations in Circular Economy and Renewable Materials, SIRRIS, 3001 Leuven, Belgium; 2 Department of Manufacturing Systems and Technologies, SIRRIS, 3600 Genk, Belgium; olivier.malek@sirris.be

**Keywords:** femtosecond laser, patterning, island-like geometry, dimensions, nanocellulose coating, hydrophobicity

## Abstract

As inspired by nature, wettability of bio-based material surfaces can be controlled by combining appropriate surface chemistries and topographies mimicking the structure of plant leaves or animals. The need for bio-based nanocellulose coatings with enhanced hydrophobic properties becomes technically relevant for extending their applications in the technological domain with better protection and lifetime of the coatings. In this work, the water repellence of spray-coated nanocellulose coatings with hydrophobically modified cellulose microfiber (mCMF coatings), or hydrophobically modified cellulose nanofiber (mCNF coatings) was enhanced after femtosecond laser patterning. In particular, the influences of different island-like pattern geometries and pattern sizes were systematically studied. The island-like patterns were experimentally created with single posts that have variable sizes of the valleys (B = 30 to 15 µm) and top surface area (T = 120 to 15 µm), resulting in good resolution of the patterns down to the size of the laser beam diameter (15 µm). Depending on the intrinsic homogeneity and porosity of sprayed mCMF and mCNF coatings, the quality and resolution of the island-like patterns is better for the mCNF coatings with thinner and more homogeneous sizes of the cellulose nanofibrils. The increase in apparent water contact angle on patterned nanocellulose coatings can be estimated from the theoretical Cassie–Baxter state of wetting and shows maximum values up to *θ*_s_ = 128° (mCMF coatings), or *θ*_s_ = 140° (mCNF coatings), for the smallest pattern sizes in parallel with minimum contact angle hysteresis of Δ*θ* = 14° (mCMF coatings), or Δ*θ* < 9° (mCNF coatings). The study demonstrated that femtosecond laser patterning technology provides high flexibility and adaptivity to create surface patterns in appropriate dimensions with enhanced hydrophobicity of nanocellulose coatings.

## 1. Introduction

Biomimetic approaches enhancing surface functionality and hydrophobicity often draw inspiration from natural surfaces, such as plant leaves, gecko feet, and butterfly wings [1,2]. These surfaces exhibit remarkable water-repellence [3], due to their unique combination of complex hierarchical structures at the micro- and nano-scale, together with the presence of wax as a hydrophobic natural substance [4]. By integrating similar patterns under controlled conditions and optimizing their geometries onto engineered biopolymer surfaces, the latter performance can be tuned [5]. The biomimetic patterning is particularly interesting for bio-based coatings [6], as it allows to develop sustainable and high-performance materials [7,8]. Since bio-based coatings are regularly soft, hydrophilic, and reactive compared to synthetic surfaces, the patterned biopolymer coatings are applicable in the medical field [9], biomedical applications [10], or microfluidics [11]. However, better protection is needed for more robust applications with enhanced durability and functionality [12], including water-repellent [13], antimicrobial [14], and antifouling properties [15]. The nanocellulose coatings have gained significant attention due to their biodegradability, mechanical strength, and tunable surface properties [16]. While traditional patterning methods of the nanocellulose surface were developed, they have certain limitations: e.g., lithography methods offer high precision but are expensive and time-consuming [17]; soft templating is cost-effective but lacks flexibility for complex patterns [18,19]; chemical etching can damage nanocellulose and may require hazardous reagents [20]; and mechanical methods may have limited precision [21,22]. In parallel with the claimed sustainable nature of sprayed nanocellulose coatings [23], the combination for additional functionalization with viable alternative processing techniques needs to be further explored.

The femtosecond laser technology has emerged as a sustainable technique for surface texturing [24], ensuring high flexibility, energy efficiency, chemical-free processing, and low waste generation [25], and it was used as a controllable way to create biomimetic surfaces [9], or superhydrophobic surfaces [26]. As the ultrashort laser pulses (10^−15^ s) locally deliver a high energy intensity over an extremely short time, it offers technological advantages over conventional laser-based texturing or nano-/picosecond laser interactions for soft and sensitive surfaces [27]. Therefore, the femtosecond lasers were well suited for the processing of non-wovens or electrospun nanofiber mats [28,29]. Due to the ultrafast interaction time, the thermal diffusion is negligible and the heat-affect zone with material degradation or damage is minimized, as demonstrated for the fiber-reinforced composites [30]. The short pulse durations allow for high precision and resolution with selective material removal. The scalability of the femtosecond laser processing for industrial application is provided through advancements in beam control, automation, and parallel processing [31], where high-throughput patterning relies on several key factors such as high-speed processing over large areas, non-contact and maskless processing, precise material interactions, parallel processing at multiple spots simultaneously, rapid beam movement, compatibility with roll-to-roll manufacturing, and minimal requirements for post processing [32,33]. In combination with biodegradable biopolymer surfaces, the femtosecond lasers allowed to directly generate high-resolution micropatterns without polymer degradation [34,35,36]. For the biopolymer films such as collagen processed at extremely high repetition rates, the local ablation was associated with swelling modifications and just local material’s removal through foaming of the irradiated area [37]. By comparing biomaterials with different water contents, thermal properties, and mechanical strengths (e.g., gelatine, chitosan, synthetic polyvinyl pyrrolidone, and biopolymer–polymer blends), the differences in size and depth of the micrometer surface profiles were obtained through the effects of beam focusing and optical aberrations [38]. The chitosan films were frequently used for femtosecond laser patterning in relation with biomedical applications, where the moderate laser conditions were applied for micromachining in the range of 150 to 350 mJ/cm^2^, resulting in pattern sizes of 10 to 50 µm [39]. In particular, the surface wettability and porosity of chitosan films irradiated in the form of grid lines in the range of 300 µm and single pulse laser-treated spots in the range of 200 µm improved intrinsic hydrophilicity and cell attachment [40], with similar results for the femtosecond laser-patterned chitosan-graphite coatings [41]. On the bacterial nanocellulose (BNC) substrates, the patterns of conductive poly(p-phenylene vinylene) were deposited by the direct femtosecond laser printing with a resolution in the order of 10 µm without material degradation [42]. Alternatively, topographies with parallel grooves featuring a 20 to 300 μm period were generated on BNC surfaces with high fidelity and reliability of the generated microstructures for controlling cell attachment [43]. In a previous study, we have demonstrated that pure nanocellulose coatings can be favorably patterned by femtosecond laser processing after investigating the influences of laser parameters [44].

In this study, the role of regular pattern geometries and sizes applied on the nanocellulose coatings through femtosecond laser ablation under known processing conditions is evaluated in order to enhance their hydrophobicity. While hypothesizing based on theoretical models that the dimensions of valley width, top surface, and height of single posts affect the maximum water contact angle on hydrophobic surfaces, it is demonstrated herein that the patterns can be practically fabricated onto sprayed nanocellulose coatings in suitable micrometer size ranges, introducing maximized hydrophobicity of the smallest pattern sizes as expected from the predicted trends.

## 2. Materials and Methods

### 2.1. Materials and Coating

The nanocellulose coatings for subsequent patterning were applied by spray coating, following the previously optimized procedures and parameters [45]. The aqueous suspensions with two hydrophobically modified nanocellulose grades were prepared, with (i) modified cellulose microfibrils (mCMFs) and (ii) modified cellulose nanofibrils (mCNFs). The hydrophobic modification of both nanocellulose grades was conducted according to a previously published protocol, including a chemical reaction with styrene-co-maleic anhydride and ammonium for the deposition of styrene-maleic anhydride nanocapsules that were filled with 70 wt.-% carnauba wax [46]. After surface modification, the suspensions of hydrophobically modified nanocellulose were diluted towards 1.5 wt.-% mCMFs and 1.5 wt.-% mCNFs. The spraying was conducted by using a handheld 1000 RP jet air spray gun (SATA GmbH & Co., Kornwestheim, Germany) under a dynamic pressure of 1.5 bar and nozzle opening of 1.6 mm. The coatings were deposited by 10 subsequent spraying layers onto borosilicate glass without intermediate drying, resulting in a total coating thickness of 6.5 ± 1 µm (mCMF coating), or 7.5 ± 1 µm (mCNF coating). The coated samples were stored under controlled lab conditions (23 °C, 50% RH) before further handling.

### 2.2. Femtosecond Laser Patterning

**Figure 1:**
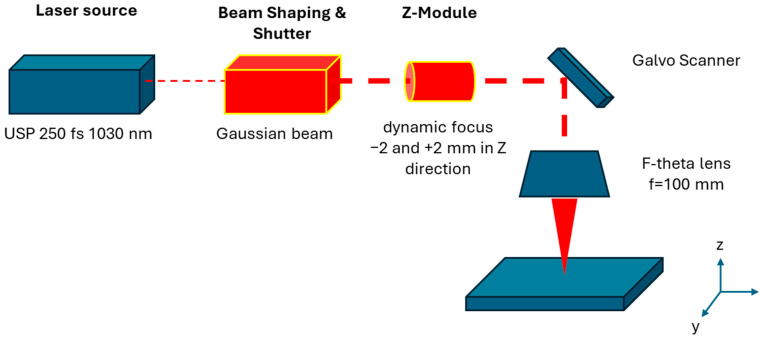
Schematic representation of the laser beam path focusing on the sample set-up used for the femtosecond laser patterning.

The patterning of the nanocellulose coatings was conducted by femtosecond laser processing on an LS5 workstation (Lasea, Liège, Belgium), equipped with a Yb-doped near-infrared laser source of 1030 nm wavelength (Satsuma HP, Amplitude Systems, Pessac, France) and operating with the ultra-short 250 fs (femtosecond) pulses under atmospheric conditions. The maximum output power of the laser is 10 W. The laser beam has a single spot size of 15 µm at the sample position, and is focused through a f-theta lens with a focusing depth of 100 µm. A schematic replication of the laser beam path between the laser source and the X, Y, Z stage controller with mounted specimen is shown in Figure 1. The operational window of applicable laser patterning parameters for given coatings was determined before [44], and known parameters were set constant in the present study for all the experimental conditions: i.e., a pulse repetition rate of 500 kHz and power setting of 39% (i.e., corresponding to a pulse energy 1.87 µJ, laser fluence 0.83 J/cm^2^), and fixed scanning speed of *v* = 4000 mm/s. The relation between pulse rate and scanning speed was set in order to obtain continuous patterns (see below), and the power setting was selected in order to obtain a good reproduction of the pattern.

**Figure 2:**
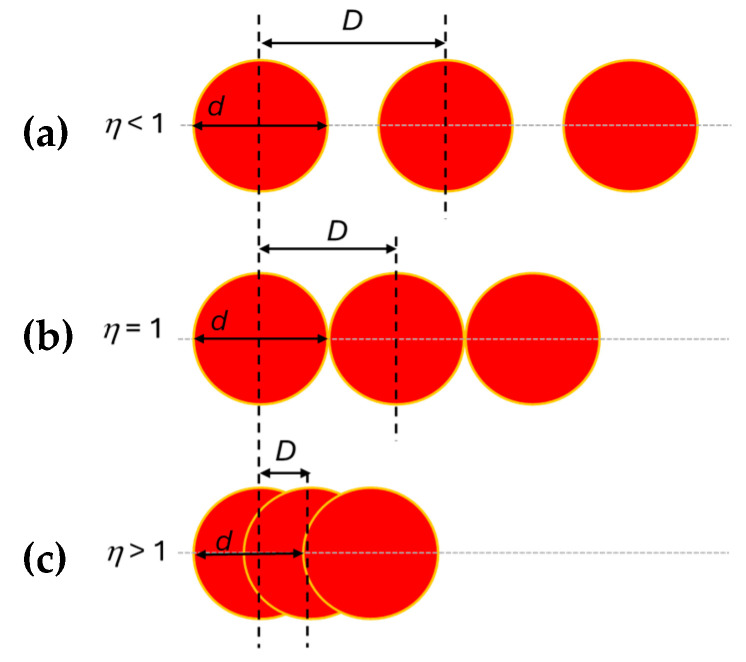
Spatial overlap between single laser pulses as determined by the net effective pulse number *η:* (**a**) No pulse overlap, *η* < 1; (**b**) Pulse touching, *η* = 1; (**c**) Pulse overlap *η* > 1.

The island-like patterns with single posts of different dimensions were created through the local removal of material by laser ablation along a programmed path of the laser beam. The size of the patterned zones on nanocellulose coatings were 1 × 1 cm^2^, corresponding to a processing time of about 3 min. The morphology of the laser ablated zone is determined by the spatial overlap of single laser pulses (Figure 2), where either the single separated laser pulses are visible as individual spots (no pulse overlap, see Figure 2a), or a continuous line of overlapping pulses is visible as a line pattern. The overlap rate of laser pulses is characterized by the selection of scanning speed and pulse repetition rate, relative to the laser spot diameter. The net effective pulse number *η* = *d*/*D*, with *d* = pulse diameter (15 μm), and *D* = distance between pulse centers, is a measure for the overlap between single laser pulses: for *d*/*D* > 1, there is an overlap in two adjacent pulses; for *d*/*D* = 1, the adjacent pulses touch; for *d*/*D* < 1, the pulses are separated for given speed. Depending on the scanning speed *v*, and pulse rate repetition frequency *f*, the pulse overlap rate *R* for a beam with spot diameter *d* can be calculated according to Equation (1),

(1) R=(d −  v/f) / d × 100

Under the present experimental settings of laser parameters with constant values of *v* = 4000 mm/s, *f* = 500 kHz, and *d* = 15 µm, a constant pulse overlap rate of *R* = 47% (*d*/*D* = 2.13) indicates a high overlap between pulses and formation of a continuous track of ablated material.

**Figure 3:**
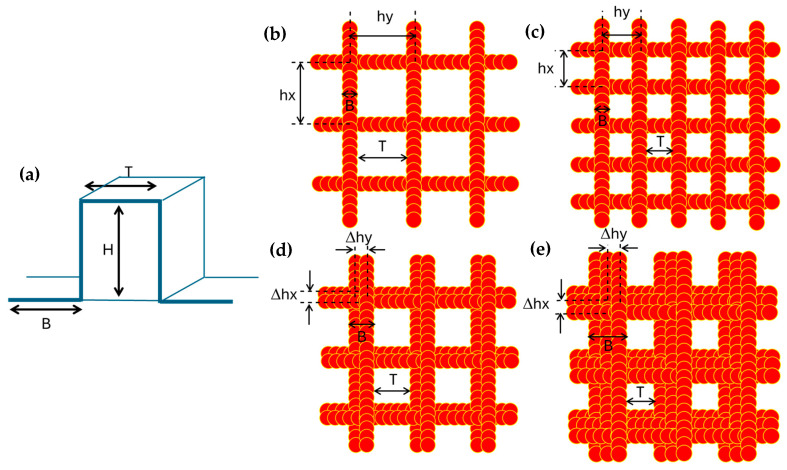
Schematic picture for creation of island-like geometrical domains with different sizes: (**a**) Representation of a single square post with dimensions H, B, T; (**b**) Path of the laser beam with spacing of the laser spot and overlap rate, with selection of hatch pitch hx, hy in respective X- and Y-direction; (**c**) Selection of smaller hatch pitch hx, hy in respective X- and Y-direction; (**d**) Selection of an offset value Δhx, Δhy for the hatch pitch during two subsequent laser ablation steps, (**e**) Selection of an offset value Δhx, Δhy for the hatch pitch during three subsequent laser ablation steps. The latter multiple laser processing steps are applied to increase B sizes and decrease T sizes.

In this work, the influence of island-like domains with different dimensions on hydrophobic properties of the spray-coated nanocellulose coatings was systematically investigated through the creation of different geometries and sizes of the single posts by laser patterning. Therefore, the coatings were experimentally patterned through laser processing, resulting in a range of pattern geometries with variations in lateral dimensions. The different sizes of valley depth B (µm), height H (µm), and top length T (µm) of the single posts were created through a variation of the hatch pitch, while multiple laser ablation steps were repeated under an offset value with different repetitions in X- and Y-direction. A schematic representation of a single post with the sizes of B, H, T is given in Figure 3a. A schematic concept on creating the domains with different dimensions by repetitive laser processing in different layers is shown in Figure 3b–e. In agreement with the laser beam diameter, the width of a single laser ablated track is 15 µm. The hatch pitches hx, hy in both plane directions and their offset values Δhx, Δhy during consecutive laser ablation layers were chosen the same in both X and Y directions, resulting in square islands with different sizes B and T. Consequently, the valley width B has a minimum theoretical width of 15 µm and the increasing sizes of B depend on the number of offset steps (Δhx, Δhy). The top length T represents the remaining top surface and depends on the selected hatch pitch (hx, hy) and offset values (Δhx, Δhy). An overview of island-like patterns with post sizes B, T that were experimentally created in this study is summarized in Table 1. The height H of the pattern is determined through topography after laser ablation (see later).

### 2.3. Surface Characterization Techniques

The 3D confocal laser scanning microscopy was conducted on a VK-X3000 microscope (Keyence Inc., Osaka, Japan) to visualize the top surface of patterned coatings by a laser interferometer picture and three-dimensional topographical view. The surface topography and profile depth is determined at an appropriate magnification of a 20× objective lens. The more detailed surface images were made by using the 50× and 150× objective lenses. The image processing was conducted with the VK Analyzer software (version 3.9.50, 2006, Keyence Inc., Osaka, Japan), including surface tilting and flatting procedures and setting appropriate offset values for roughness determination.

The contact angles of sessile water droplets were measured on an OCA50 contact angle device (Dataphyiscs Instruments GmbH, Filderstadt, Germany), after deposition of a 3 µL volume droplet of de-ionized water. A statistical variation on contact angles was obtained as the standard deviation of 5 independent measurements. The contact angle hysteresis was obtained from dynamic contact angle measurements with de-ionized water: the advancing contact angle was determined when progressively increasing the droplet volume to 7 µL; and the receding contact angles were determined when reducing the droplet volume and retraction of the droplet contact line over the surface. The droplet geometries were fit by a tangent line at both the left and right side of the droplet. Measurements on highly hydrophobic surfaces were possible by using a dispensing needle with non-sticky PTFE coating for easy detachment and transfer of the pending water droplets onto the patterned hydrophobic surfaces.

## 3. Results

### 3.1. Substrates of Sprayed Hydrophobic Nanocellulose Coatings

**Figure 4:**
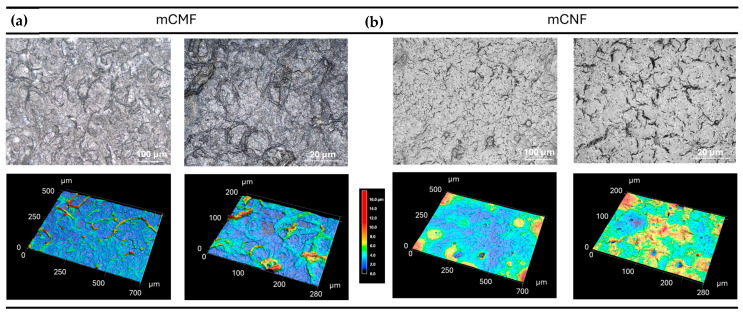
Confocal laser microscopy of sprayed nanocellulose coatings before laser patterning: (**a**) Hydrophobic cellulose microfiber coatings (mCMF); (**b**) Hydrophobic cellulose nanofiber coatings (mCNF), with laser image (**top**) and 3D topographical image (**bottom**) in two magnifications (50×, 150× objective lens). The z-scale range applies to all topographical images.

The spraying of cellulose micro- or nanofibers from aqueous suspension is a rapid and novel process for the fabrication of either freestanding wet films [47] or coatings [23]. In particular, the spraying of hydrophobically modified nanocellulose suspensions has been recently evaluated [48]. Because the spraying application provides a contactless deposition of the coating layer on the solid surface and a contour coating is formed, the surface topography and morphology of the solid surface does not influence the coating process and coating weight [49]. The spraying of CNF on single textile fibers and fabrics with a hierarchical structure [50] or paper substrates was already developed for the deposition of dense barrier coatings on porous substrates [51,52]. It has been more recently implemented to coat also other substrates such as impermeable glass or stainless steel [53]. While the adhesion onto smooth surfaces might be problematic, here, we used a sand-blasted glass substrate with Sa (average surface roughness) = 0.10 ± 0.02 µm in order to deposit a most homogeneous coating with good adherence to the substrate through mechanical interlocking, without the need for additional chemical surface activation. The latter substrates were chosen from a practical standpoint fulfilling the needs of the present work without the need for further optimization of adhesion. The morphology of sprayed mCMF and mCNF coatings is illustrated in Figure 4, as delivered for the laser patterning. The surfaces are characterized by the morphology of the respective nanocellulose fibers.

The fibrillated celluloses are known to form dense coatings owing to the intimate contact between the individual cellulose fibrils after drying. Although there is a relatively low concentration of the aqueous nanocellulose suspensions, a closed film is formed after multiple spraying steps according to our previous experiences [45]. The drying process of the wet coatings can likely be conducted in an air oven at a temperature of 105 °C or in a laminar flow chamber or fume hood with a constant flow of air under standard laboratory practice for a longer time until the constant weight of the coating [49]. For present conditions, optimized drying conditions were set at 60 °C for 30 min in a hot air oven, as determined by the constant weight reduction of the coating through complete evaporation of the water. The corresponding thickness of the fully dried coatings as determined by topographical measurements are 6.55 ± 0.50 µm (mCMF coatings) and 7.35 µm ± 0.30 µm (mCNF coatings). The apparent densities of the coatings determined from weight and thickness measurements are 1.45 g/cm^3^ (mCMF coatings) and 1.53 g/cm^3^ (mCNF coatings).

The sprayed mCMF and mCNF coatings have a compact structure, with a more heterogeneous surface for the hydrophobic microfibrillated cellulose (mCMF) coatings and smoother surface for the hydrophobic nanofibrillated cellulose (mCNF) coatings. Depending on the production process for the cellulose fibrillation, it is known that the higher degree of fibrillation through mechanical processing does not only produce the smaller fibrils, but it also reduces the variation and heterogeneity in fibrillar sizes [53]. The latter variations in morphology of the single cellulose fibrils is obviously transferred into the more homogeneous morphology and higher density of mCNF coatings than mCMF coatings. Consequently, the surface roughness parameters for sprayed mCMF and mCNF coatings are highly different depending on the fineness of the nanocellulose fibrils, as quantified in Table 2. The values for the surface roughness parameters Sa (average roughness), Sz (maximum height), and Sv (maximum valley depth) are measured on three independent locations under standard conditions with an objective lens 20×. The differences in roughness are mainly attributed to protrusions of microfibrillar aggregates at the top surface. As an intrinsic property of the sprayed nanocellulose coatings, the surface roughness values are indicatively to be taken into account and challenging for patterning by laser processing. Although the focusing depth of the laser beam is relatively higher than the surface roughness and it favorably covers the surface heterogeneities, the interactions between the laser beam and the surface may introduce local light scattering effects. In particular for polymers, and soft surfaces in general, the creation of regular patterns under femtosecond laser pulses and their periodic structure may be a function of the surface roughness and/or presence of local surface heterogeneities [54]. For the non-wovens, the fineness of the patterned microstructures was not limited by the Gaussian laser beam profiles, but rather determined by effects such as light scattering [55]. The challenge for micropatterning of rough and porous media with well-defined geometries by ultrashort-pulsed lasers requires experimental conditioning of the laser parameters and target materials [56], as shown in our present study.

### 3.2. Laser Patterning of Sprayed Hydrophobic Nanocellulose Coatings

**Figure 5:**
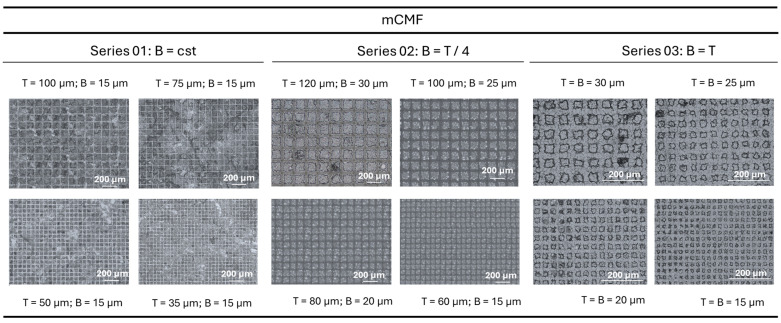
Confocal laser interferometry of island-like domains on mCMF coatings created by femtosecond laser patterning with different geometrical dimensions of individual posts with selected T, B sizes (series according to Table 1).

**Figure 6:**
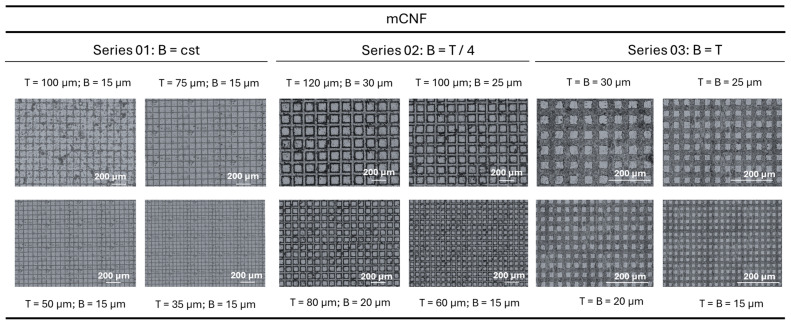
Confocal laser interferometry of island-like domains on mCNF coatings created by femtosecond laser patterning with different geometrical dimensions of individual posts with selected T, B sizes (series according to Table 1).

The experimental results of laser-patterned mCMF coatings with different lateral sizes of the top surface (T) and valley widths (B) of the single posts forming island-like domains are shown in Figure 5, as obtained under constant laser processing conditions as mentioned above. The patterns with similar sizes of B and T were also created on mCNF coatings by laser patterning under the same conditions, as shown in Figure 6. The surface morphologies are best visualized with high contrast through the laser intensity images (optical micrographs not clear due to optical transparency of the coatings) at given constant magnification, in order to indicate the long-range reproducibility of the patterns.

While the preliminary evaluations on influences of laser processing conditions (power 37 to 45%, pulse repetition rate 60, 255, or 500 kHz) for similar (non-modified) nanocellulose coatings were presented before and the operational window of processing parameters was determined through a design-of-experiments [44], the selected conditions for 39% power, 500 kHz pulse rate (corresponding to a calculated laser fluence of 0.83 J/cm2) remain valid and were used as optimized processing conditions in present cases for both mCMF and mCNF coatings. It was demonstrated that the different sizes of the patterns were successfully created with good resolution in the micrometer size ranges: the minimum size of B = 15 µm as created by a single laser ablation step in X- and Y-direction corresponds well with the dimensions of the laser beam and can be reproduced in combination with the selection of different hatch pitches (see previous Figure 3). The differences in replication and precision of the patterns can be noticed for either mCMF or mCNF coatings. In parallel with the mentioned differences in roughness and homogeneity of sprayed mCMF and mCNF coatings (see previous Figure 4), the patterns can be better reproduced on the smoother mCNF coatings while some defects and irregularities may occur for mCMF coatings. In general, the interactions of the laser light beam with natural fibers or textile surfaces are very complex and were theoretically analyzed in a comprehensive work [57]: while the light interaction with single fibers might be known, the absorption, transmission, and reflection of the incident laser beam with woven textile structures and/or non-woven fiber mats mainly depends on the relation between laser wavelength versus density and local interactions at the fiber cross-overs. Alternatively, the light beam interactions with nanofiber mats are dominated by scattering effects that are influenced by the internal structure of the nanofiber network and mainly dominated by the nanofiber size [58]. Therefore, the small size of mCNF nanofibers relative to the laser beam wavelength is favorable to reduce light scattering effects.

**Figure 7:**
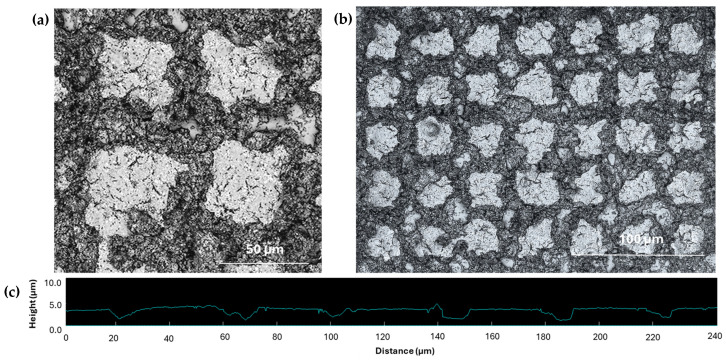
Detailed microscopic observation of femtosecond laser-patterned mCMF coatings with an example of single island-like domains: (**a**) Local microscopy of island-like domains with sizes B = 15 µm, T = 50 µm (series 01), laser intensity image; (**b**) Long-range detail and island-like domains with sizes B = T = 20 µm (series 03), optical image; (**c**) Height profile over several posts on mCMF coatings.

**Figure 8:**
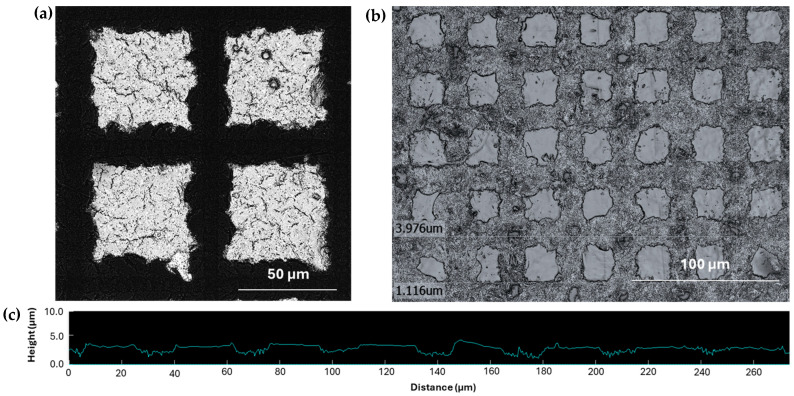
Detailed microscopic observation of femtosecond laser-patterned mCNF coatings with an example of single island-like domains: (**a**) Local microscopy of island-like domains with sizes B = 15 µm, T = 50 µm (series 01), laser intensity image; (**b**) Long-range detail and island-like domains with sizes B = T = 20 µm (series 03), optical image; (**c**) Height profile over several posts on mCNF coatings.

The detailed microscopic images of the individual posts on mCMF coatings (Figure 7) and mCNF coatings (Figure 8) are compared. For mCMF coatings, the long fiber entanglements may hinder a precise reproduction of the borders and interspaces of the island-patterned coating. The more irregular fiber protrusions and higher surface roughness for mCMF coatings introduce local defects in the laser patterns due to irregular ablation and likely strong physical or mechanical interlocking between the single fibrils. As another reason, mCMF coatings are also more porous and have consequently weaker mechanical properties owing to the heterogeneous fiber sizes and less controlled fiber interactions, which might lead to a local collapse of the patterns at weak interface borders. The better surface homogeneity and higher density of mCNF coatings as demonstrated before, can be expected in parallel with the thin fibrillar sizes and theoretically stronger fiber interactions, resulting in a better quality of the island patterns. The better quality of the sprayed mCNF coatings compared to mCMF coatings also resulted in the better mechanical properties of the mCNF coatings, as reported before [45].

**Figure 9:**
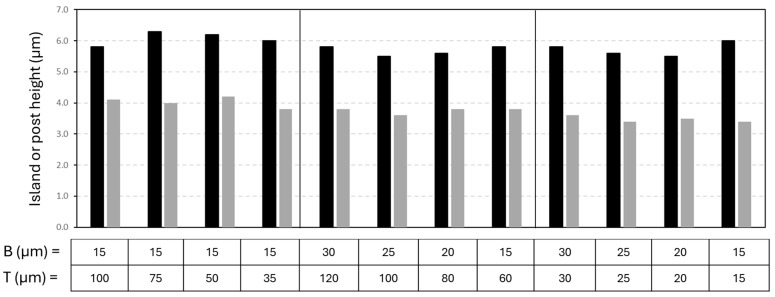
Height dimensions (H) of individual posts on island-like geometrical patterns for mCMF coatings (black bar) and mCNF coatings (gray bar), as experimentally determined from profilometry measurements.

Based on the profilometry measurements taken over the island-like patterns (see Figure 7c and Figure 8c), the averaged height values of the single posts are summarized in Figure 9. According to the topographical information, the height of the posts is at around 6.0 ± 0.5 µm (mCMF coatings) or 3.6 ± 0.5 µm (mCNF coatings). Independently of the pattern sizes, there is a relatively good agreement in the height of the posts for the different geometries on either mCNF or mCMF coatings, taking into account the heterogeneity and relatively high surface roughness of the coatings. It indeed indicates that the depth of laser ablation is mainly determined by the laser parameters. The height of posts on mCMF coatings is significantly higher as the coatings are more porous and obviously more easily ablated, as compared to the denser mCNF coatings that are more difficultly ablated under the same laser ablation parameters. The height of the posts remains smaller than the coating thickness for mCNF coatings (<7.35 µm ± 0.30 µm), while it becomes in the range of the thickness for mCMF coatings (≈6.55 ± 0.50 µm). A possible reduction of post height on mCMF coatings could not be realized under mild laser ablation conditions (e.g., reduced power, low repetition rates), as the island geometries became not clearly resolved. Therefore, the replication of island-like domains depends on the relation between laser ablation conditions and substrate characteristics, which was experimentally optimized. Overall, the geometry of island-like patterns in an appropriate range of micrometer sizes can be successfully controlled and reproduced on soft nanocellulose coatings, depending on the intrinsic coating properties relative to the laser processing parameters. The experimental generation of surfaces with reproducible island-like domains and controllable dimensions in the micrometer size range as presented, is crucial in further controlling the wetting properties of the hydrophobic nanocellulose coatings.

**Figure 10:**
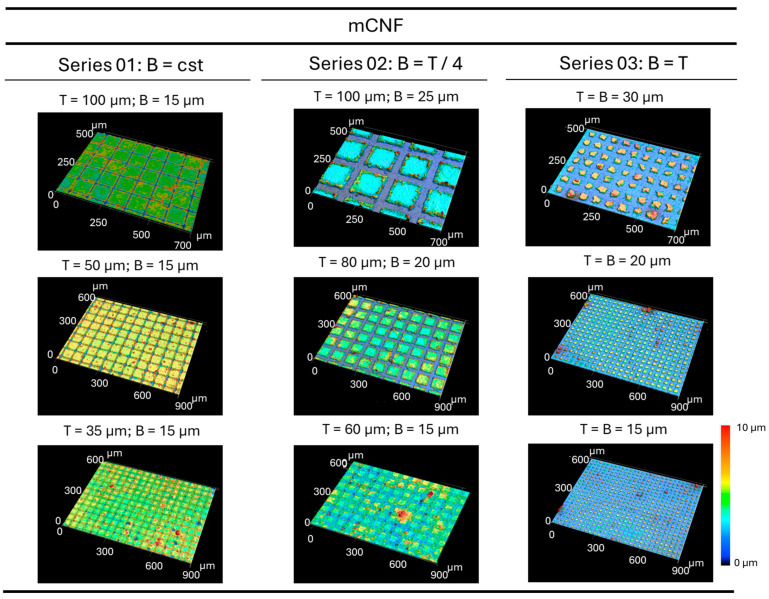
Three-dimensional topographical images of island-like domains on mCNF coatings created by femtosecond laser patterning with different geometrical dimensions of individual posts according to the selected T, B sizes (series according to Table 1). Same color applies for all Z scales.

An example of three-dimensional topographic images of the island-like domains with different sizes on mCNF coatings (i.e., selected coating for best resolution of the patterns) is shown in Figure 10. The features include a broad variety in sizes and interspaces of the islands (see Table 1), and also confirm the good adherence of the domains on the glass substrate after laser ablation. The latter is in agreement with the height of the individual posts relative to the thickness of the sprayed coatings, confirming that the glass substrate does not become exposed and the posts are created within the nanocellulose coating layer itself (see Figure 9). The defect patterns with removal of the domains from the substrate were observed under the more intense laser ablation conditions at high power (e.g., 40%) and 500 kHz pulse repetition rate (i.e., pulse energy 2.07 µJ, laser fluence 0.91 J/cm^2^).

### 3.3. Hydrophobicity of Nanocellulose Coatings with Island-like Domains

#### 3.3.1. Theoretical Reference Values of Water Contact Angles

The wetting state of water droplets on heterogeneous surfaces, particularly those that are rough or porous, can theoretically be described by the Cassie–Baxter model. It can be applied as a prediction of the surface wetting based on static contact angles and has been demonstrated before to be applicable for femtosecond laser-ablated steel and aluminum surfaces [59], or elastomeric siloxane [60]. While the theoretical model was initially applied on hard solid substrates, the applicability under present conditions with more heterogeneous nanocellulose coatings is further evaluated as below.

Following the Cassie–Baxter model, the apparent contact angle of a water droplet on a regularly roughened surface is influenced by the presence of air pockets trapped between the droplet and substrate. This results in the water droplet sitting on a heterogeneous surface made up of both solid material and air. In this state, the water droplet does not fully wet the surface while it rests on the tops of the surface roughness peaks, with air pockets trapped underneath. The Cassie–Baxter wetting state on rough surfaces consequently leads to a higher water contact angle as appearing on a smooth surface, making the surface more hydrophobic. By increasing the roughness and reducing the contact area between the water droplet and the solid surface, the contact angle can consequently be significantly increased. The Cassie–Baxter Equation (2), or in a reduced form in Equation (3), can be used to calculated the apparent contact angle, as follows [61]:

(2) $$\cos\theta_{c\;}=f_{s}\cos\theta_{s}-f_{v}$$

(3) $$\cos\theta_{c\;}=f_{s}(\cos\theta_{s}+1)-1$$

where:

*θ_c_* is the apparent contact angle on a rough, patterned surface;
*f_S_* is the fraction of the solid surface area in direct contact with the liquid;
*θ_s_* is the equilibrium contact angle on the non-patterned solid surface;
*f_v_* is the fraction of the surface area where the liquid is in contact with air (with *f_s_* + *f_v_* = 1).

Although the Cassie–Baxter model predicts a water contact angle by using surface energy and simplified roughness factors (*f_s_*, *f_v_*), important limitations should be considered. The proposed basic form can only be correct for the case of flat-topped post geometries where the liquid droplet touches the tops of the surface without much penetration [62], e.g., when additional hydrophobic or water-repellent coating is present in combination with the surface texture. For these reasons, the model is valid only in some range of wetting states and numerous follow-up studies have been conducted to complement these models. Alternatively, the effects of surface roughness on surface hydrophobicity were more explicitly studied, as both the vertical-perspective roughness factors as well as the horizontal-perspective roughness factors considerably affects the hydrophobicity. Therefore, the roughness parameter *r*_1_ was introduced in order to correct the surface contact ratio for roughness effects, resulting in a corrected Cassie–Baxter equation as given in Equation (4) [63,64]. For given geometry of island-like domains with single post dimensions H, B, T as defined before, the values can be calculated as *r*_1_ = (T + B)/(2H + T + B) and *f_s_* = T/(T + B). The surface roughness ratio then represents a ratio of projected versus total surface area, and *f_s_* is the fraction of solid wetted surface area.

(4) $$\cos\theta_{c\;}={r_{1}f}_{s}\cos\theta_{s}-(1-f_{s})$$

**Figure 11:**
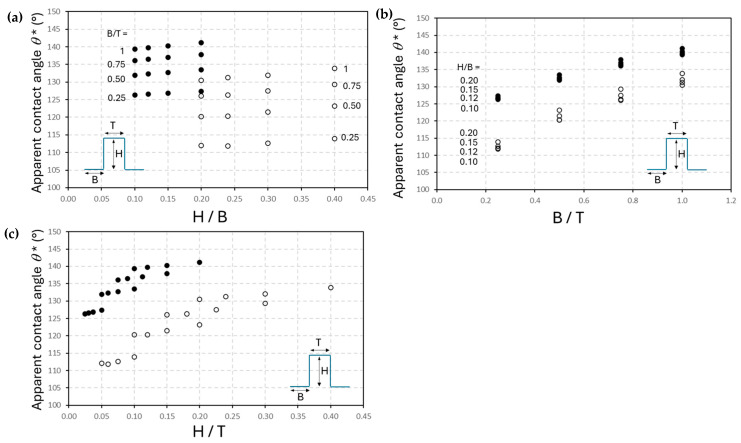
Theoretical model calculations following the Cassie–Baxter equation of apparent contact angles θ* to be expected on mCMF coatings (open symbols, o) and mCNF coatings (closed symbols, ●), after island-like patterning with different H, B, T sizes of individual posts (see insets). The calculated values for θ* are plotted as a function of (**a**) H/B ratio, (**b**) B/T ratio, and (**c**) H/T ratio.

For the island-like geometries with different post sizes H, T, and B, the theoretical influences of the named dimensions on the apparent contact angles were calculated following the latter Equation (4). The theoretical values are plotted in Figure 11 for both mCMF coatings (*θ_s_* = 98°) and mCNF coatings (*θ_s_* = 118°), visualizing the different trends on apparent contact angles depending on the characteristic dimensional ratios of the posts, e.g., H/B, B/T, and H/T. The variations in ratio H/B has little effect on augmenting the apparent contact angles, while the ratios B/T and H/T have a larger effect through the effective influence of the contacting surface area T. It is in agreement with previous calculations for solid silicon substrates [65], where results demonstrated that solid fraction (T) is the key determinant parameter, with a decreasing solid fraction leading to higher contact angles. It was also proven on polymer substrates that with the introduction of square pillars, the influences of pillar height on hydrophobicity are inferior [66]. In contrast, changes in edge-to-edge spacing and micro-post shape/arrangement have relatively minor effects. From these theoretical trends, a maximized hydrophobicity on patterned nanocellulose coatings is predicted for the smaller dimensions of top surface area T and wider dimensions of valleys B (e.g., high B/T), in particular for the geometrical patterns with B =T in series 03 (see Table 2).

#### 3.3.2. Experimental Water Contact Angles

**Figure 12:**
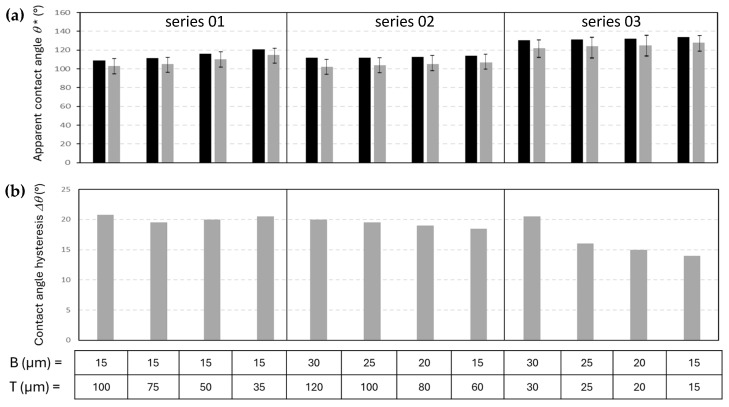
Hydrophobic properties of patterned mCMF coatings with island-like geometries of different B, T size (series according to Table 1): (**a**) Static water contact angles θ* (theoretical calculations in black bar, experimental value in gray bar); (**b**) Contact angle hysteresis of water Δ*θ* (difference in advancing and receding contact angles).

**Figure 13:**
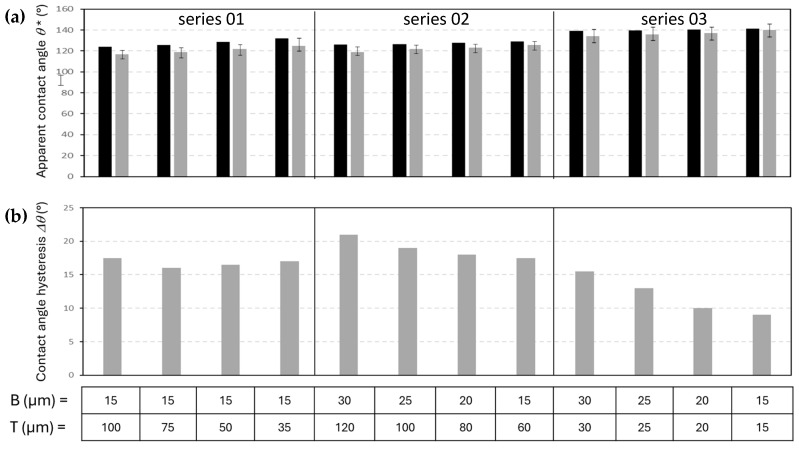
Hydrophobic properties of patterned mCNF coatings with island-like geometries of different B, T size (series according to Table 1): (**a**) Static water contact angles θ* (theoretical calculations in black bar, experimental value in gray bar); (**b**) Contact angle hysteresis of water Δ*θ* (difference in advancing and receding contact angles).

The hydrophobicity of the patterned nanocellulose coatings was experimentally determined by measuring the static water contact angles *θ^∗^*, and calculating the contact angle hysteresis Δ*θ* as the differences between advancing and receding contact angles. The experimental results in comparison with the previously calculated theoretical values for the different island-like pattern geometries (i.e., series 01, 02, 03, see Table 1), are summarized for patterned mCMF coatings (Figure 12) and mCNF coatings (Figure 13).

The experimental values of static water contact angles (Figure 12a and Figure 13a) follow very well the expected trends according to theoretical calculations based on the Cassie–Baxter equation, where experimental values (within a statistical error of about ±3°) are systematically few degrees below the theoretical values. The latter is evident, as the real surfaces may have some more local variations in morphology and resulting contact angles. The static water contact angles on patterned mCNF coatings are higher as compared to patterned mCMF coatings, in agreement with the initial static water contact angle differences between both coatings and the better replication of the island-like geometries on mCNF coatings, as discussed before. For both mCMF and mCNF coatings, the highest static water contact angles occur for geometrical patterns with B = T (series 03) and smallest dimensions of the posts with B = T = 15 µm: e.g., with maximum values θ* = 128° for patterned mCMF coatings and θ* = 140° for patterned mCNF coatings. A decreasing trend for the apparent contact angles with increasing size of the posts was also noticed before in experimental and theoretical approaches for patterned metal surfaces with hydrophobic coating [67], and is confirmed by our tests.

The values for contact angle hysteresis (Figure 12b and Figure 13b) are known to be dominated by the surface roughness, and seem to be lower on smooth mCNF coatings compared to rougher mCMF coatings. In parallel with the geometries of island-like domains resulting in highest static water contact angles, the smallest values for contact angle hysteresis are: e.g., Δ*θ* = 14° mCMF coatings and Δ*θ* = 9° for mCNF coatings, occurring for the same geometrical patterns with B = T (series 03) and smallest dimensions of the posts with B = T = 15 µm. The particularly high values of receding water contact angles mainly occur on rough surfaces, as the retraction of the water droplet is hindered through pinning of the retracting droplet contact line near local roughness peaks. Overall,, the contact angle hysteresis remains high for the present nanocellulose coatings and they are limited in providing a further reduction of contact angle hysteresis. Therefore, the range of contact angles and contact angle hysteresis is applicable for coatings with enhanced hydrophobicity, while superhydrophobic properties under present conditions may occur for coatings having only a very specific combination of a nanocellulose type and pattern geometry. In summary, the best performance for an optimized geometrical pattern on mCNF coatings is obtained with water contact angles of Δ*θ* = 140° and contact angle hysteresis of Δ*θ* = 9°, approaching the superhydrophobic range. The lowest contact angle hysteresis for nanocellulose coatings would infer the use of a silanized or perfluorinated surface modification of the cellulose fibrils [68,69]: for the latter conditions, the superhydrophobic properties introduce water contact angles of 162° and sliding angle < 10°.

**Figure 14:**
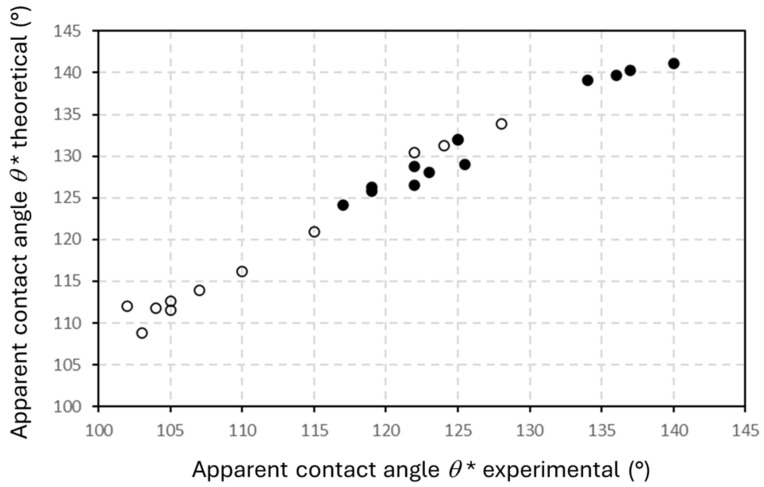
Comparative graph for values of apparent water contact angles, including theoretical values (Cassie–Baxter model) versus experimental values on patterned mCMF coatings (open symbols, o) and patterned mCNF coatings (closed symbols, ●).

In conclusion, the experimental water contact angles and theoretical calculations following the Cassie–Baxter model are comparable (Figure 14). The predictions based on the given island-like geometries provide reliable estimates for the experimental contact angles. From these observations, the flexibility for creating island-like geometries with suitable micron-scale dimensions through femtosecond laser ablation provides a suitable technology for maximizing hydrophobicity of nanocellulose coatings, as expected.

## 4. Conclusions

This study successfully demonstrated how different geometries and sizes of regular island-like patterns were applied by femtosecond laser patterning onto modified nanocellulose coatings for boosting hydrophobic properties. As it was hypothesized from a theoretical Cassie–Baxter wetting state, it can be expected that the smaller micrometer-sized patterns enhance the hydrophobicity of the coatings. A good agreement between theoretical predictions and experimental results for water contact angles on patterned coatings of modified cellulose microfibrils (mCMFs) and cellulose nanofibrils (mCNFs) was confirmed. Through variations in micrometer dimensions and ratios of B, T, H sizes for individual surface posts, the influences of different pattern geometries was systematically investigated, concluding that the smallest sizes B = T = 15 µm provide highest hydrophobicity on mCNF coatings with an increase of *θ*^∗^ = 118°, Δ*θ* = 25° (non-patterned) towards *θ*^∗^ = 140°, Δ*θ* = 9° (patterned).

From a practical point of view, the femtosecond laser patterning of soft nanocellulose coatings offers reproducible patterns that can be produced within a small processing window of laser parameters, with smallest sizes for the width of valleys (B) and top surfaces (T) in the range of the laser beam diameter, and depth of the laser ablation (H) mainly determined by the laser energy relative to the coating properties. The patterning of smooth mCNF coatings is more reproducible compared to CMF coatings owing to various reasons discussed (e.g., density, porosity, mechanical properties).

The presented technology for micromachining of soft biopolymer coatings offers a flexible method for improving functionality by selection of appropriate pattern geometries. Although the improvement in hydrophobicity is a most essential property for bio-based coatings, the other specific pattern geometries may be considered to improve other characteristics such as anti-microbial or lubrication properties in the future.

## Figures and Tables

**Table 1:** Overview for lateral sizes of single posts (geometries of island-like domains with B and T sizes, as in Figure 2b), experimentally created by femtosecond laser patterning of modified nanocellulose coatings.

Sample Series	B Size (µm)	T Size (µm)
Series 01: B = cst	15	100
	15	75
	15	50
	15	35
Series 02: B = T/4	120	30
	100	25
	80	20
	60	15
Series 03: B = T	30	30
	25	25
	20	20
	15	15

**Table 2:** Surface roughness parameters (Sa, average roughness; Sz, maximum height; Sv maximum valley) of sprayed nanocellulose coatings as delivered for laser patterning. Measurements from three independent images per coating type.

Coating Type	Sa (µm)	Sz (µm)	Sv (µm)
mCMF	1.39 µm 1.93 µm 1.54 µm	18.0 µm 25.1 µm 19.8 µm	4.28 µm 6.06 µm 4.78 µm
mCNF	1.01 µm 1.13 µm 1.08 µm	12.5 µm 17.9 µm 16.5 µm	2.89 µm 3.09 µm 3.01 µm

## Data Availability

Dataset available on request from the authors.
